# Fewer invited talks by women in evolutionary biology symposia

**DOI:** 10.1111/jeb.12198

**Published:** 2013-06-20

**Authors:** J Schroeder, H L Dugdale, R Radersma, M Hinsch, D M Buehler, J Saul, L Porter, A Liker, I De Cauwer, P J Johnson, A W Santure, A S Griffin, E Bolund, L Ross, T J Webb, P G D Feulner, I Winney, M Szulkin, J Komdeur, M A Versteegh, C K Hemelrijk, E I Svensson, H Edwards, M Karlsson, S A West, E L B Barrett, D S Richardson, V van den Brink, J H Wimpenny, S A Ellwood, M Rees, K D Matson, A Charmantier, N dos Remedios, N A Schneider, C Teplitsky, W F Laurance, R K Butlin, N P C Horrocks

**Affiliations:** 1 Department of Animal and Plant Sciences, University of SheffieldSheffield, UK; 2 Evolutionary Biology, Max Planck Institute for OrnithologySeewiesen, Germany; 3 Behavioural Ecology and Self-Organization, University of GroningenGroningen, The Netherlands; 4 Theoretical Biology, University of GroningenGroningen, The Netherlands; 5 Department of Zoology, Edward Grey Institute, University of OxfordOxford, UK; 6 Department of Ecology and Evolutionary Biology, University of TorontoToronto, ON, Canada; 7 Department of Natural History, Royal Ontario MuseumToronto, ON, Canada; 8 Department of Philosophy, University of SheffieldSheffield, UK; 9 Department of Limnology, University of PannoniaVeszprém, Hungary; 10 Laboratoire de Génétique et Evolution des Populations Végétales, UMR CNRS 8198Lille, France; 11 Department of Zoology, Wildlife Conservation Research Unit, University of OxfordOxford, UK; 12 Department of Zoology, University of OxfordOxford, UK; 13 Evolutionary Ecology, Max-Planck Institute for Evolutionary BiologyPloen, Germany; 14 Centre d'Ecologie Fonctionnelle et Evolutive, UMR 5175 Campus CNRSMontpellier, France; 15 Animal Ecology, University of GroningenGroningen, The Netherlands; 16 Department of Biology, Evolutionary Ecology Unit, Lund UniversityLund, Sweden; 17 Department of Biology, MEMEG, Lund UniversityLund, Sweden; 18 School of Biological Sciences, University of East AngliaNorwich, UK; 19 Department of Ecology and Evolution, University of Lausanne; 20 Department of Biology and Biochemistry, University of BathBath, UK; 21 Department of Ecology, Swedish University of Agricultural SciencesUppsala, Sweden; 22 CERSP, UMR 7204 CNRS / MNHN /UPMCParis, France; 23 School of Marine and Tropical Biology, James Cook UniversityCairns, QLD 4878, Australia; 24 Department of Zoology, University of CambridgeCambridge, UK

**Keywords:** career ladder progression, conference presenters, discrimination, evolutionary biology, gender difference, implicit bias, invited speakers, leaky pipeline, scientific visibility, sex ratios

## Abstract

Lower visibility of female scientists, compared to male scientists, is a potential reason for the under-representation of women among senior academic ranks. Visibility in the scientific community stems partly from presenting research as an invited speaker at organized meetings. We analysed the sex ratio of presenters at the European Society for Evolutionary Biology (ESEB) Congress 2011, where all abstract submissions were accepted for presentation. Women were under-represented among invited speakers at symposia (15% women) compared to all presenters (46%), regular oral presenters (41%) and plenary speakers (25%). At the ESEB congresses in 2001–2011, 9–23% of invited speakers were women. This under-representation of women is partly attributable to a larger proportion of women, than men, declining invitations: in 2011, 50% of women declined an invitation to speak compared to 26% of men. We expect invited speakers to be scientists from top ranked institutions or authors of recent papers in high-impact journals. Considering all invited speakers (including declined invitations), 23% were women. This was lower than the baseline sex ratios of early-mid career stage scientists, but was similar to senior scientists and authors that have published in high-impact journals. High-quality science by women therefore has low exposure at international meetings, which will constrain Evolutionary Biology from reaching its full potential. We wish to highlight the wider implications of turning down invitations to speak, and encourage conference organizers to implement steps to increase acceptance rates of invited talks.

## Introduction

In the sciences, there are fewer women than men at graduate level and even fewer among senior academic positions (European Commission, 2011). In 2006, 36% of EU PhD graduates in Science and Engineering were women, reducing slightly to 33% among post-doctoral researchers (Grade C), then falling dramatically to 11% of the senior academic ranks (Grade A; European Commission, 2011; figure II.3.13). The ‘leaky pipeline’ is often used as a metaphor for the loss of women transgressing to more senior positions in scientific academia, and the synthesis of empirical evidence is important to understand the causes underlying the leak (COSEPUP, 2007; European Commission, 2011; Dugdale *et al*., 2011).

Reaching a senior academic position requires academic success. An important and widely used metric of academic success is the production of many widely-cited publications, but academics can also raise their profiles by giving invited seminars and networking at universities and international conferences (van den Brink, 2011). Assuming the work is well presented, exciting and scientifically sound, these activities positively increase the profile or visibility of a researcher. This can induce a self-reinforcing feedback loop: increased visibility signals quality (i.e. Damschen *et al*., 2005), and researchers with increased visibility are expected to be more likely to be invited back as a guest or plenary speaker, which further enhances visibility. If the first step to gaining visibility is impeded, the positive feedback loop will not occur or will be less effective.

We hypothesize that because the scientific achievements of women may be less visible than the achievements of men (Thelwall *et al*., 2006; Fernandez *et al*., 2009), female scientists may be overlooked more often for invitations to talk. If this is true, we expect the sex ratio of invited speakers to be biased towards males, even after accounting for career stage and the population sex ratio of the research field. The sex ratio of speakers at a symposium can also depend on the genders of the symposium organizers, with fewer women speaking in male-only organized symposia (Isbell *et al*., 2012). We therefore expect that symposia organized only by men will have fewer female invited speakers than symposia that have at least one female organizer.

To test these two hypotheses, we analysed data on invited speakers from six biannual congresses of the European Society for Evolutionary Biology (ESEB; 2001–2011), and in a more detailed case study, all contributions of the last ESEB congress in 2011 in Tübingen, Germany, which accepted all abstract submissions. In 2011, submissions that were not accepted as a conventional contribution were accepted in the form of an essence poster, a smaller version of a normal poster. We tested for gender differences between all presenters, and for each presentation category. It is also necessary to compare these sex ratios to baseline sex ratios of populations of researchers in the tested career stages, since sex ratios differ between career stages (European Commission, 2011). Detected gender differences may also be related to methods of speaker selection. For example, speaker selection may be associated with reputation, which is often measured by citation metrics that can also be affected by gender (Symonds *et al*., 2006). One may therefore argue that sex ratios may differ between a group of top quality scientists that merit invitation and all others. We therefore compared the sex ratios of invited speakers with the sex ratios of baseline populations of scientists who merit invitations to speak, through demonstration of their excellence in science.

Our motivation for this research was to detect gender differences that could lead to a disproportionate visibility of men vs. women in Evolutionary Biology, while controlling for career stage and presumed research quality. It is of course unfair to highlight one particular scientific society, and we wish to point out that we could have selected other societies. For example, gender differences have been noted in Ecology (Holt & Webb, 2007) and in Primatology (Isbell *et al*., 2012) meetings. Our intention is not to apportion blame, nor to judge any person or group of persons involved in the selection procedure. Quite the opposite: we want to draw attention to the processes that may cause the scientific community to miss out on a substantial proportion of high-quality science, which in turn may slow progress in Evolutionary Biology.

## Materials and methods

### Data selection

At the 13th ESEB congress, all 1023 abstract submissions for talks and posters were accepted thanks to the introduction of essence posters (a smaller version of a regular poster, www.eseb2011.de, accessed November 2011). According to the ESEB website, the congress had 337 slots for talks, which included slots for 8 plenary and 66 specifically invited speakers that carry a higher prestige status. Each symposium was allocated a number of talk slots according to the proportion of submissions they received; hence, acceptance rates for submitted talks (roughly 20–30%; www.eseb2011.de) were independent of a symposium's topic. Additionally, approximately 500 submissions were accepted as a regular poster, and the remaining contributors were offered essence posters.

The congress programme detailed 1022 contributions, of which 73 were invited (8 plenary speakers and 65 invited speakers) and 949 were applied for (276 regular speakers, 479 regular posters and 194 essence posters). We determined the gender of the first author through meeting them in person, or by their first name given in the list of participants in the congress guides. From here on, when we write ‘presenter’ or ‘speaker’, we refer to the first author of a contribution of any format, as listed in the congress guide. We followed the same procedure to assess the gender of the organizers of the 30 symposia.

We contacted all symposia organizers to request the number and gender of all of the speakers that they invited and of those that declined their invitation. We also present the sex ratios of the invited and plenary speakers deduced from the congress guides for the ESEB congresses in 2001, 2003, 2005, 2007 and 2009.

Further methodological details are provided in the Supplementary Methods S1.

### Baseline populations

Sex ratios change with career stage; thus, sex ratios of invited speakers need to be compared to sex ratios of the baseline population of scientists at a given career stage (essentially the pool of scientists that could be invited to speak). Ideally, our analyses would take the age and seniority of invited speakers into account. Since the qualities that define scientists who merit invitations to speak at an international conference are debatable, we provide several metrics for comparison. As a first metric, we suggest that invited speakers could be leaders in their field, so we compared the sex ratio of invited speakers with the faculty sex ratios from the Evolutionary Biology departments at the world top-10 universities for the Life Sciences (Times Higher Education University Ranking 2010–2011http://www.timeshighereducation.co.uk/world-university-rankings/2010-2011/life-sciences.html, accessed May 2012). Our decision to choose only the top-10 universities was somewhat arbitrary. An analysis of only the top-10 European universities led to similar conclusions (see Supplementary Methods S1). We determined the sex ratio for the following three career stages: (1) Professors, Readers and Full Professors (henceforth: ‘Professors’), (2) Associate Professors, Senior Lecturers and Lecturers (‘Lecturers’) and (3) Assistant Professors and Fellows (‘Fellows’).

Another reason a speaker might be selected as an invited speaker is that he or she has made an important research discovery. We therefore used the search engines of the two highest-impact journals to calculate the sex ratios of first and last authors of primary research articles in these top-tier journals, *Nature* and *Science*. Finally, for comparison, we also present the overall sex ratios of faculty in biosciences in the UK, and the sex ratio of faculty in science and engineering across the EU (HESA, 2012).

The statistical methods are detailed in the Supplementary Methods S1.

## Results

**Figure 1:**
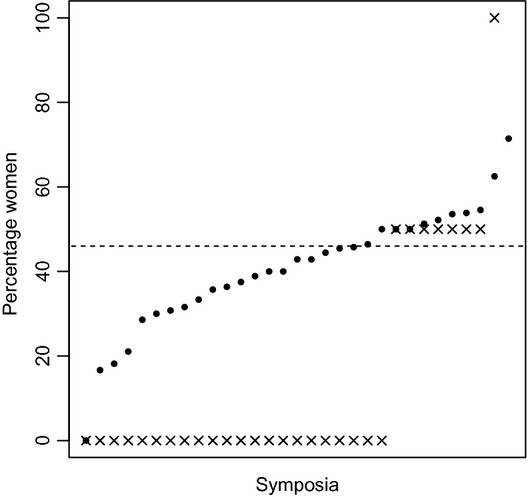
The percentage of presenters that were women, per symposium, at the ESEB 2011 congress (ordered by sex ratio; percentage women). The dashed line represents the sex ratio among all presenters at ESEB 2011 (46%). Bullets represent the sex ratio among all presenters in each symposium at ESEB 2011, including: essence posters, regular posters, regular talks and invited talks (per 31 symposia, ordered by sex ratio). Crosses represent the sex ratio among invited speakers at ESEB 2011 (per 30 symposia).

**Figure 2:**
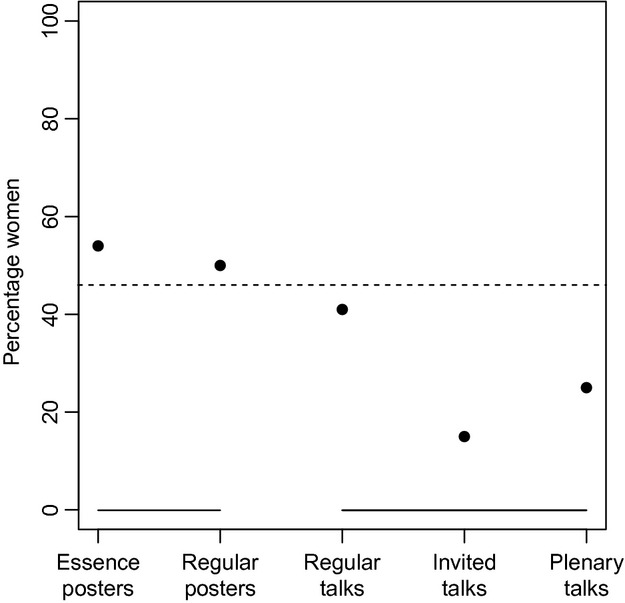
Sex ratios (percentage women) of the five formats of presentations at ESEB 2011. The dashed line shows the 46% sex ratio of all presenters. Solid lines indicate category affiliation.

Women accounted for 46% of all presenters (including all oral and all poster categories) at ESEB 2011; however, within symposia, the percentage of female presenters varied from 0–71% (Fig.1). A lower percentage of women presented talks than posters (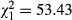, *P *<* *0.001; Fig.2); women presented 54% of all essence posters, 50% of all regular posters, 41% of regular talks (where the submitted abstracts were peer selected), 15% of invited talks (excluding plenary speakers) and 25% of plenary talks (Fig.2).

The sex ratio of the plenary speakers (6 men and 2 women) did not differ significantly from the sex ratio of all other oral presenters (odds ratio: 0.40, 95% confidence interval [95CI]: 0.04 – 2.24, *P *=* *0.30), or from the sex ratio of all regular speakers (odds ratio: 0.47, 95CI: 0.05 – 2.72, *P *=* *0.48). The sex ratio of invited speakers was biased towards males compared to all other presenters (Fig.1, 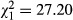, *P *<* *0.001) and compared to all regular speakers (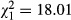, *P *<* *0.001).

Although 23% of all initially invited speakers (including those that declined) were women, only 15% of the realized invited speakers were women. This reduction was because 50% of invited women declined talks compared to 26% of invited men (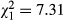, *P *=* *0.007).

Only 10 of the 30 symposia organizing committees contained women, and only 18% of symposia organizers were women (53 male, 12 female). This differed from the sex ratio of all presenters (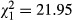, *P *<* *0.001) and of regular speakers (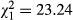, *P *<* *0.001). There was no association between the presence or absence of female organizers and the respective sex ratio of their invited speakers (estimate ± SE* *=* *−0.65 ± 1.21; *z*_1,28_* *=* *−0.54, *P *=* *0.59), contrasting with the findings of Isbell *et al*. (2012).

**Figure 3:**
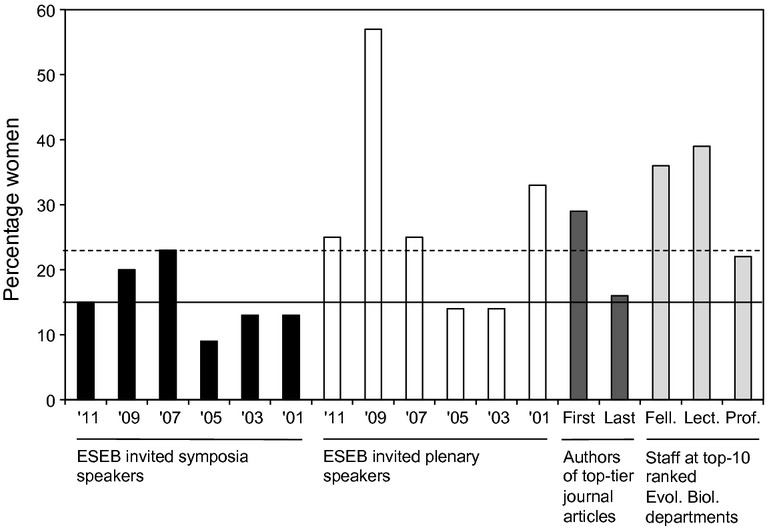
The percentage of invited speakers that were women, in symposia (black bars) and plenaries (white bars), at ESEB congresses in 2001–2011, in comparison with the percentage of women in baseline populations of first and last authors in top-tier journals (dark grey bars), and faculty members (light grey bars; Fell. = Fellows, Lect. = Lecturers, Prof. = Professors). Horizontal lines under the *x*-axis indicate the specific category groupings that the bars belong to. The horizontal continuous line in the plot indicates the sex ratio among the realized invited speakers at ESEB 2011, and the dashed line indicates the sex ratio among all initially invited speakers at ESEB 2011, including those who declined to participate.

At past ESEB congresses (2001–2011), the sex ratios of realized invited speakers varied between 9% and 23% (Fig.3). The sex ratios of plenary speakers varied from 14% to 57% (Fig.3). The faculty sex ratio at the top-10 universities in the Life Sciences was: (1) Professors: 22% ± 1.7% SE (range: 15–32%), (2) Lecturers: 39% ± 8.3% SE (range: 0–100%); and (3) Fellows: 36% ± 7.3% SE (range: 0–86%). On average, in the top-tier journals 29% of first authors and 16% of last authors of evolutionary research articles were women.

**Figure 4:**
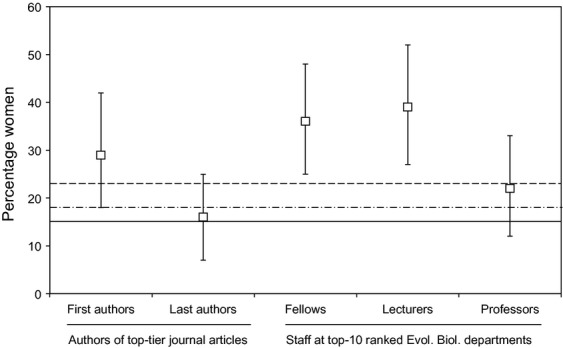
The percentage of invited speakers that were women, selected by randomizations from baseline populations of authors in top-tier journals (first and last authors) and faculty members (error bars = 95% confidence intervals). Horizontal lines under the *x*-axis indicate the specific category groupings that the data points belong to. The horizontal continuous line in the plot indicates the sex ratio among the realized invited speakers at ESEB 2011, the dash-dotted line indicates the sex ratio among symposium organizers at ESEB 2011 and the dashed line indicates the sex ratio among all initially invited speakers at ESEB 2011, including those who declined to participate.

Randomizations showed that the sex ratio of realized invited speakers (15% women) was lower than baseline populations of early–mid career stage scientists (including first authors in top-tier journals), but similar to senior scientists (Professor and last authors in top-tier journals; (Fig.4)). However, the 23% of initially invited speakers who were women (and of whom a larger proportion of women than men declined to speak) was lower than baseline populations of early–mid career stage scientists (Lecturers & Fellows) but did not differ from Professors or authors in top-tier journals (Fig.4). Testing just against the faculty baselines, the 18% of symposium organizers that were women was lower than Fellows and Lecturers but not Professors (Fig.4). The career stages of the ESEB 2011 invited speakers at the time of invitation (2010) were 46% Professors, 33% Lecturers and 20% Fellows. According to our baseline sex ratios, we would therefore expect 20 women among the invited speakers, instead of the 10 observed.

## Discussion

The sex ratio of realized invited speakers at ESEB congresses was male biased, compared to several baseline populations. Previous studies reporting gender biases in academia have not statistically compared their data with baseline populations while considering the effects of career stage or age, which may contribute to the bias. Gender bias however cannot be documented and addressed without knowledge of the sex ratio of the baseline population from which the studied data were drawn and examination of the underlying mechanisms. We show that the sex ratio bias of realized invited speakers was primarily due to fewer men than women declining invitations. We discuss three potential and not mutually exclusive mechanisms leading to fewer women invited speakers: (1) the pool of scientists that could be invited to speak contains fewer women than men, for example due to the ‘leaky pipeline’; (2) women turned down invitations more often than men; and (3) there was a bias for selecting men as invited speakers.

(1) Potentially, there are fewer women, than men, that could be invited to speak. In the face of strong sex ratio differences in many science and engineering fields (e.g. Smyth & McArdle, 2004), it is encouraging that the overall sex ratio of scientists presenting their work at the 2011 ESEB congress was nearly equal. Moreover, there was no strong deviation from this overall sex ratio compared to presenters of both poster categories and regular talks. However, invited talks were strongly male biased, contrasting with most of our baseline populations.

These figures of sex ratios among faculty are comparable to faculty in the UK Biosciences in 2010–2011, where women made up 46% of Lecturers or Junior Fellows, 32% of Senior Lecturers and Readers and 19% of Professors (HESA, 2012). Additionally, 32% of fellows from the Natural Environment Research Council in the UK, a major funding agency for research in Evolutionary Biology, were women (2008–09 to 2010–11; NERC, 2011). While these numbers can only give us a rough estimation of the expected sex ratio of the baseline population of scientists that could be invited to speak, they all have similar or higher sex ratios than the invited speakers at the ESEB congress 2011.

The only group of scientists with similarly few women as the realized invited speakers at ESEB congresses were the last authors in two top-tier publications (16%) and Professors (22%). Our author baselines are conservative given that gender differences may occur in publication rates (Braisher *et al*., 2005; Symonds *et al*., 2006). Nevertheless, our comparisons with baseline populations imply that we miss a significant proportion of high-quality Evolutionary Biology research during invited talks, and that this research does not get the visibility in our field that it potentially deserves. This may impact on the careers of female scientists, reducing their visibility (e.g. for promotion), which in turn decreases the number of female role models in Evolutionary Biology.

Preferably, one should correct our analyses for the age or seniority of the invited speakers. This requires a more in-depth analysis, which would be a valuable future avenue of research. Overall, however, it seems unlikely, given our baseline populations, that the sex ratio among potential invitees is much lower than 20%. We therefore do not believe that there were fewer women who were potentially eligible to give invited talks. Rather other factors are contributing to the under-representation of female invited symposia speakers, but not plenary speakers.

(2) We found that a larger proportion of women than men turned down invitations to speak at ESEB 2011. The process of selecting invited speakers was relatively unbiased: 23% of all initially invited speakers were women. This was similar to most of our baseline sex ratios, except for Lecturers and Fellows in the top-10 Evolutionary Biology departments, which were significantly higher. This shows that, by our measures, the number of women invited initially to ESEB 2011 was not biased; however, women were more likely to turn down an invitation than men, contributing to the low realized number of invited female speakers. Many reasons may underlie this, for example compared to men, women might find it more difficult to travel to meetings (potentially due to childcare or carer duties, Mason & Goulden, 2004), self-promote less (Moss-Racusin & Rudman, 2010), and have a lower perception of their success (Rammstedt & Rammsayer, 2001; Sieverding, 2003), specifically of their scientific ability (Dugdale *et al*., 2011; Laurance *et al*., 2011). While childcare is increasingly available at larger congresses, this was not the case at ESEB 2011. Further research is required to assess whether unavailable or expensive childcare while travelling causes women to reject invitations. Gender differences in grant awards occur (Bornmann *et al*., 2007); therefore, invited women may have less travel funding than men which may contribute to them declining more often. If the rates of declines by women were an outlier at ESEB 2011, other mechanisms must have been at play to explain the similar low percentage of invited female speakers at the other ESEB congresses. It is interesting to note, though, that the sex ratios among ESEB plenary speakers (Fig.3) were in all cases higher than the sex ratio of invited speakers.

(3) A third mechanism that could lead to fewer female invited speakers could be implicit bias, a known cause for women being at a disadvantage when climbing the career ladder. Both males and females subconsciously treat and perceive women and men differently, even if they are equally skilled and experienced (Valian, 1999; COSEPUP, 2007). People tend to assign fame more often to men than women (Banaji & Greenwald, 1995; Damschen *et al*., 2005). Seeing mainly male invited speakers may reinforce an expectation that matches ‘invited speaker’ with ‘male’, leading to fewer women being invited (Valian, 1999).

A large body of evidence highlights the existence of implicit bias against women in science (Steinpreis, 1999; Miller & Chamberlin, 2000; Trix & Psenka, 2003; Schmader *et al*., 2007), and it has been proposed as the underlying cause of the low numbers of women chairing sessions at British Ecological Society meetings (Holt & Webb, 2007). Bias against women in the evaluation of grants appears to have been eliminated in Sweden (Sandström & Hällsten, 2008), but globally, men are more likely to win grants, especially post-doctoral fellowships (Bornmann *et al*., 2007).

However, it is reassuring that the overall sex ratio of initially invited speakers (23% including those that declined) at ESEB 2011 was comparable to most of the sex ratios of our baseline populations. Additionally, the presence or absence of female organizers within a symposium did not influence the sex ratio of their invited speakers. This suggests that evolutionary biologists do not harbour much implicit bias against female scientists. Still, as the realized sex ratios are distorted, we are exposed to fewer women presenting excellent research, which can generate a feedback loop based on visibility and the perception of a gender-biased impression of high-quality research, independent of merit.

In summary, women were more likely to decline invitations, and this was an important factor explaining the low proportion of women presenting invited talks at ESEB 2011. If this finding can be generalized, then we may be missing a substantial proportion of high-quality research. Dissemination of knowledge of the underlying problems is crucial to provide a long-term solution. Therefore, congress and symposia organizers and invitees alike should be aware of the higher decline rate of women, and of the risks of unconscious bias when selecting invited speakers. It is also important to increase awareness among organizers as well as invitees that contributions by an appropriate number of woman scientists are important beyond the research content, as it increases the visibility of female scientists in general. In the long term, higher exposure to more female scientific leaders will help fight implicit bias (Dasgupta & Asgari, 2004; Kang & Banaji, 2006; Asgari *et al*., 2010; Stout *et al*., 2011), provide us with a more comprehensive overview of the high-quality research in our field, and help to patch the ‘leaky pipeline’.
