# Supplementary material for: Fewer invited talks by women in evolutionary biology symposia
Source: J Evol Biol. 2013 Jun 20;26(9):2063–9. doi: 10.1111/jeb.12198 (PMC4293461; doi:10.1111/jeb.12198)
Supplement: Supplementary file 1 — Supplementary Methods S1. [file jeb0026-2063-sd1.pdf]

Fewer invited talks by women in evolutionary biology symposia

Julia Schroeder[1,2]*, Hannah L. Dugdale[1,3,4]*, Reinder Radersma[5], Martin Hinsch[3,4], Deborah M. Buehler[6,7], Jennifer Saul[8], Lindsey Porter[8], András Liker[1,9], Isabelle De Cauwer[1,10], Paul J. Johnson[11], Anna W. Santure[1], Ashleigh S. Griffin[12], Elisabeth Bolund[1], Laura Ross[12], Thomas J. Webb[1], Philine G. D. Feulner[13], Isabel Winney[1], Marta Szulkin[12,14], Jan Komdeur[3], Maaike A. Versteegh[15], Charlotte K. Hemelrijk[3], Erik I. Svensson[16], Hannah Edwards[1], Maria Karlsson[17], Stuart A. West[12], Emma L. B. Barrett[18], David S. Richardson[18], Valentijn van den Brink[19], Joanna H. Wimpenny[1], Stephen A. Ellwood[11], Mark Rees[1], Kevin D. Matson[15], Anne Charmantier[14], Natalie dos Remedios[1,20], Nicole A. Schneider[21], Celine Teplitsky[22], William F. Laurance[23], Roger K. Butlin[1], Nicholas P. C. Horrocks[15,24]

* equal authorship

[1] Department of Animal and Plant Sciences, University of Sheffield, Sheffield, UK.

[2] Evolutionary Biology, Max Planck Institute for Ornithology, Seewiesen, Germany.

[3] Behavioural Ecology and Self-Organization, University of Groningen, Groningen, Netherlands

[4] Theoretical Biology, University of Groningen, Groningen, Netherlands

[5] Edward Grey Institute,Department of Zoology, University of Oxford, Oxford, UK.

[6] Department of Ecology and Evolutionary Biology, University of Toronto, Toronto, Ontario. Canada.

[7] Department of Natural History, Royal Ontario Museum, Toronto, Ontario.

[8] Department of Philosophy, University of Sheffield, Sheffield, UK.

[9] Department of Limnology, University of Pannonia, Veszprém, Hungary

[10] Laboratoire de Génétique et Evolution des Populations Végétales, UMR CNRS 8198, Lille, France

[11] Wildlife Conservation Research Unit, Department of Zoology, University of Oxford, Oxford, UK.

[12] Department of Zoology, University of Oxford, Oxford, UK.

[13] Evolutionary Ecology, Max-Planck Institute for Evolutionary Biology, Ploen, Germany

[14] Centre d'Ecologie Fonctionnelle et Evolutive, UMR 5175 Campus CNRS, Montpellier, France

[15] Animal Ecology, University of Groningen, Groningen, Netherlands

[16] Evolutionary Ecology Unit, Department of Biology, Lund University, Lund, Sweden

[17] MEMEG, Department of Biology, Lund University, Lund, Sweden

[18] School of Biological Sciences, University of East Anglia, Norwich, UK

[19] Department of Ecology and Evolution, University of Lausanne

[20] Department of Biology and Biochemistry, University of Bath, Bath, UK

[21] Department of Ecology, Swedish University of Agricultural Sciences, Uppsala, Sweden

[22] CERSP, UMR 7204 CNRS / MNHN /UPMC, Paris, France

[23] School of Marine and Tropical Biology, James Cook University, Cairns, QLD 4878, Australia

[24] Department of Zoology, University of Cambridge, Cambridge, UK

This document contains Supplementary Methods S1.

(S1) Supplementary Methods:

Sex ratios of presenters and symposia organisers

The number of invited speakers differed slightly between the website totals (www.eseb2011.de, accessed November 2011) and the printed congress guide. Here, we used the printed copy of the congress guide that was issued at registration. We determined the gender of the first author through meeting them in person, or by their first name given in the list of participants in the congress guide. If the gender of the name was ambiguous, and/or we did not meet or know the scientist in question, we used the first author's name and email address to look them up on their departmental website. We were able to unambiguously determine the gender of all speakers, but not of 45 of the poster presenters (19 essence posters and 26 regular posters). These presenters of unknown gender were excluded from all following analyses.

ESEB funded the conference fees, but not travel costs, of two invited speakers per symposium; however, symposium organisers could invite additional speakers if they secured outside funding. Twenty-five symposia had two invited speakers, two had four, two had three, and one had one. One symposium, with three invited speakers, was a merger of three symposium proposals. This symposium had not documented all changes to their invited speakers due to the mergers, and we excluded this from our analyses of declined talks.

The deadline for calls for ESEB symposia are generally at least a year in advance, at which time potential organisers must have contacted their invited speakers to confirm their availability. Successful symposium proposals are then selected by a committee. Most ESEB 2011 symposia had two organisers (one had one organiser, 26 had two, two had three, and one had six since it was a merger of three symposium proposals).

Baseline populations - faculty

We compared the sex ratio of invited speakers with the faculty sex ratios from the Evolutionary Biology departments at the world top-10 universities for the Life Sciences (Times Higher Education University Ranking 2010–2011, http://www.timeshighereducation.co.uk/world-university-rankings/2010-2011/life-sciences.html; accessed May 2012) to the most accurate level that each institutional website allowed. We excluded the John Hopkins University (Rank 8), as their Biology Department did not have a distinct Evolutionary Biology group; instead we added Imperial College London (Rank 11) to complete the top-10 Evolutionary Biology departments. We used the following departments and universities: MIT, Biology; Harvard University, Human Evolutionary Biology and Molecular Cellular Biology; Stanford, Department of Biology; University of Oxford, Zoology; University of Yale, Ecology and Evolutionary Biology; University of Cambridge, Zoology; Imperial College London, Division of Ecology and Evolution; Princeton University, Ecology and Evolutionary Biology; and, University College London, Research Department of Genetics, Evolution and Environment.

Our decision to choose only the top-10 universities was somewhat arbitrary. We therefore also assembled data of Evolutionary Biology faculty in the widest sense for European Universities only (the top 10 in the same ranking, Cambridge, Oxford, Imperial, UCL, ETH Zürich, Edinburgh, LMU Munich, Utrecht University, Uppsala University and Ghent University, accessed May 2013). The numbers are similar: Professors 24% (SE = 2%), Lecturers 29% (SE = 5%), and Fellows 40% (SE = 4%).

Baseline populations – authors in top-tier journals

In Evolutionary Biology, the first author is usually the one who wrote the text and did most of the work, while the last author is usually the primary investigator who secured funding and supervised the work. We used the journal-specific search engines to select relevant papers. We used the search engine of the journal *Science* to search for original research contributions between January 2010 and January 2012 with the keywords 'evolution', 'evolved' or 'evolution*' in the title. A similar search was not possible directly on the site of the journal *Nature*. We therefore used their search mechanism by subject and selected all articles and letters published in *Nature* under the subject category 'Evolution' between January 2010 and January 2012. We disregarded any results from the Earth Sciences. Then, we determined the sex ratio of the first and last authors of these articles, excluding articles authored by consortia.

Statistical analyses

All statistical analyses were performed in R.2.15.1 (R Development Core Team, 2011). We compared the sex ratios (presented as percentage women) of poster presenters (regular posters and essence posters) and oral presenters (plenary speakers, invited speakers, regular speakers). Plenary speakers, as defined in the ESEB 2011 congress guide, include the presidential address and the invited presentation by the John Maynard Smith prize winner, but the statistical results did not change qualitatively when we excluded these. We tested the sex ratio differences using a χ^2 test, with Yate's correction for continuity (Mantel & Greenhouse, 1968).

We then compared the sex ratio of invited speakers with the sex ratio of all other presenters, and the sex ratio of plenary speakers with that of all other presenters. Since the χ^2 test is prone to type II errors for small sample-sizes (Crawley, 2007),

when expected counts were less than 5 we applied Fisher's exact test to test for a deviation from an odds ratio of 1 between female and male speakers in the tested categories. One could argue that oral presenters represent a different group of scientists compared with poster presenters (Isbell *et al.,* 2012) because peer-review deemed their abstracts of higher quality and/or to reflect topics of higher interest than those of the poster presenters. Hence, they may represent more experienced scientists, meaning that comparisons between both groups (oral and poster presenters) might not be valid. Additional, gender differences in self-promotion (Moss-Racusin & Rudman, 2010) may result in fewer women applying for oral presentations. While we do not test for a difference in scientific quality or self-selection between posters and oral presentations, we acknowledge that these could bias our analyses. We therefore tested for differences in the sex ratio of invited versus regular oral presenters, and of plenary versus regular oral speakers, assuming that peer review facilitates an equally high quality of all oral presentations.

Some invited speakers declined invitations to speak. We therefore tested for a difference in the sex ratios of invited speakers that declined or accepted an invitation to speak (hereafter termed: ‘initially invited’ [i.e. including declines] and ‘realised invited’ [i.e. excluding declines] speakers) using the χ^2 test. 20 women were invited initially; 10 accepted and 10 declined. Whereas 68 men were initially invited; 50 accepted and 18 declined.

The sex ratio of speakers at a symposium can depend on the gender of the symposium organiser (Isbell *et al.,* 2012). We first tested whether the sex ratio of the symposium organisers differed from that of all presenters and of regular presenters. We then tested for an association between the presence and absence of women among the organisers of a symposium and the sex ratio of their invited speakers (listed in the

congress guide), using a generalised linear model with binomial error structure and logit link.

If bias occurs when selecting invited speakers, we would expect the sex ratio of invited speakers to differ from the baseline populations of scientists who could qualify as invited speakers at ESEB. We used the rbinom function in R to compare the sex ratio of both the realised and initially invited speakers from all 30 symposia, to those from 10,000 randomisations. The randomisations facilitated comparison of the sex ratios of our baseline populations with that of the invited speakers, accounting for the fact that each symposium was limited to two ESEB-funded invited speakers. In each randomisation, two invited speakers were randomly selected 30 times, using the sex ratios of the three career stages (Professors, Lecturers and Fellows) of faculty members of Evolutionary Biology departments (from the world top-10 rankings in Life Sciences) and authors of current high-impact journals (i.e. first and last authors of primary research articles in *Nature* and *Science*). We also tested the sex ratios of the symposium organisers against the baseline sex ratios of different career stages of faculty members.

References

Crawley, M.J. 2007. *The R Book*. Wiley & Sons, Chichester.

Isbell, L., Young, P.T. & Harcourt, A.H. 2012. Stag parties linger: Continued gender bias in a female-rich scientific discipline. *PLoS ONE* **7**: e49682.

Mantel, N. & Greenhouse, S.W. 1968. What is the continuity correction? *Am. Stat.* 22: 27–30.

Moss-Racusin, C.A. & Rudman, L.A. 2010. Disruptions in women's self-promotion: The backlash avoidance model. *Psychol. Women Q.* 34: 186–202

R Development Core Team 2011. *R: A language and environment for statistical computing*. R Foundation for Statistical Computing, Vienna, Austria.
